# Participatory methods on the recording of traditional knowledge about medicinal plants in Atlantic forest, Ubatuba, São Paulo, Brazil

**DOI:** 10.1371/journal.pone.0232288

**Published:** 2020-05-07

**Authors:** Thamara Sauini, Viviane Stern da Fonseca-Kruel, Priscila Baptistela Yazbek, Priscila Matta, Fernando Cassas, Crenilda da Cruz, Eduardo Hortal Pereira Barretto, Maria Alice dos Santos, Maria Angelica Silva Gomes, Ricardo José Francischetti Garcia, Sumiko Honda, Luiz Felipe Domingues Passero, Bruno Esteves Conde, Eliana Rodrigues

**Affiliations:** 1 Center for Ethnobotanical and Ethnopharmacological Studies (CEE), Department of Environmental Sciences, Universidade Federal de São Paulo (UNIFESP), São Paulo, Brazil; 2 Jardim Botânico do Rio de Janeiro, Rio de Janeiro, Brazil; 3 Amerindian Studies Center, Universidade de São Paulo (CEstA-USP), São Paulo, Brazil; 4 Associação dos Remanescentes de Quilombo do Cambury, Ubatuba, São Paulo, Brazil; 5 Herbário Municipal (PMSP), Secretaria Municipal do Verde do Meio Ambiente da Prefeitura do Município de São Paulo, São Paulo, Brazil; 6 Institute of Biosciences, São Paulo State University (UNESP), São Vicente, São Paulo, Brazil; University Lyon 1 Faculty of Dental Medicine, FRANCE

## Abstract

**Introduction:**

Ethnobotanical studies that include participatory methods aim to engage residents in different steps to promote the strengthening and perpetuation of local culture, and empowerment in making decisions about the use of available environmental resources. Thus, the aim of this project was to perform an ethnobotanical survey based on traditional knowledge of medicinal plants with the active participation of residents living in Bairro do Cambury, Ubatuba, São Paulo State, Brazil.

**Materials and methods:**

During meetings held between the researchers and community members, locally used plants were regarded as an important means for preserving local knowledge for future generations. Some residents showed interest in participating as local partners, and training courses for collecting ethnobotanical data were offered. Local partners and researchers from São Paulo Federal University (Universidade Federal de São Paulo) utilized ethnobotanical methods to select and interview the specialists in medicinal plants for 80 days between 2016 and 2018. Data on plant use were recorded, and plants were collected and deposited in two herbaria. Furthermore, participant observation and fieldwork diaries were used by the researchers, aiding the data analysis.

**Results:**

Three local partners participated in objective definitions, data collection, analysis and publication. Nine local specialists were interviewed by the local partners and indicated the use of 82 plant species in 90 recipes for 55 therapeutic uses. These uses were grouped into 12 categories. In addition, a video and booklet were created.

**Conclusions:**

Data obtained during participatory research show that training local communities in the registration of their own knowledge is feasible and necessary since they register knowledge based on local perceptions, as well as valuing knowledge and approaching the current discussion about intellectual property is a global concern.

## Introduction

The participatory method in ethnobotany has recently gained the attention of different authors [1–6] since the active participation of residents in ethnobotanical study phases can promote, among other things, their empowerment decision about the use of environment available resources. Works performed by Medley and Kalibo and Kalibo and Kimberly [7, 8] described and demonstrated ways to develop participatory methodologies during ethnobotanical research, which can assist in the understanding of local knowledge. Furthermore, the importance of local members training as consultants and partners has been emphasized, since their participation in resource management improves natural resource conservation.

Etkin and Ticktin [4] emphasized the need for the participation of local members in all investigation process steps from initial design to data analysis. However, there have been few published studies in which the research has involved local residents from study design to data recording and analysis. The present survey, together with that developed by Hitziger et al. [9] and another recently published by our team among Quilombolas living in Quilombo da Fazenda [10], brings concrete and pioneering examples of a new approach focusing on the development of large-scale cooperative research in ethnobotany. The last study was conducted by the same team as this study and with the same methods; compared to the methods used by the Hitziger et al. [9], it shows a higher degree of involvement than observed in many ethnobotany studies, since community members actively participated in all the stages of development: from objective definitions, to plants collection and registration and the return of information collected to the community.

The purpose of ethnobotany based on participatory research comes from classical studies, which routinely involved simple reports by ethnobotanists on the traditional use of plants within different cultures. However, the evolution of this area has meant that other subjects must be considered, such as local development, conservation and sustainability [11]. Therefore, ethnobotany can be considered an applied science; thus, all of the acquired data can be used to promote local development and conservation of the environment [11, 12]. In this sense, including the local inhabitants in such studies as members of the ethnobotanical study can be more effective for local development.

The Atlantic forest is one of the main five biomes in Brazil, and it hosts several traditional communities. It is considered one of the most biodiverse biomes in the world, with approximately 20 thousand species of plants, of which approximately eight thousand are endemic, including many endangered species, and only approximately 7% of the original forest cover remains [13]. The Atlantic forest is considered a world biodiversity hotspot and one of the most endangered areas on the planet [14, 15].

Although different studies of ethnobotany have been performed in the Atlantic forest among traditional communities [16–19], none of them applied a participatory methodology. In this context, the present study focused on the following: 1) surveying medicinal plants used by residents living in Bairro do Cambury (BC) and 2) testing a pioneering participatory method in which residents acted as local partners, idealizing survey objectives, selecting interviewees, recording their knowledge, collecting plants that they commonly use, analysing data obtained and participating as co-authors of study publications.

## Materials and methods

### Ethics statement

This project was initiated in April 2015, and it received Brazilian legal permissions: 1) to access the area of the Serra do Mar State Park (COTEC no. 260108–009.510/2015); 2) to collect plants and access Serra da Bocaina National Park (SIBIO no. 51199-1/2015); 3) to obtain prior informed consent and access traditional knowledge (SISGEN no. A648D14); and 4) to be conducted by the Universidade Federal de São Paulo, (Research Ethics Committee approval no. 028525/2016). The residents authorized the use of their images in the video. It took 17 months to obtain all of these permits, and data collection began only after all of the permits were obtained, in September 2016.

### Study area

**Fig 1:**
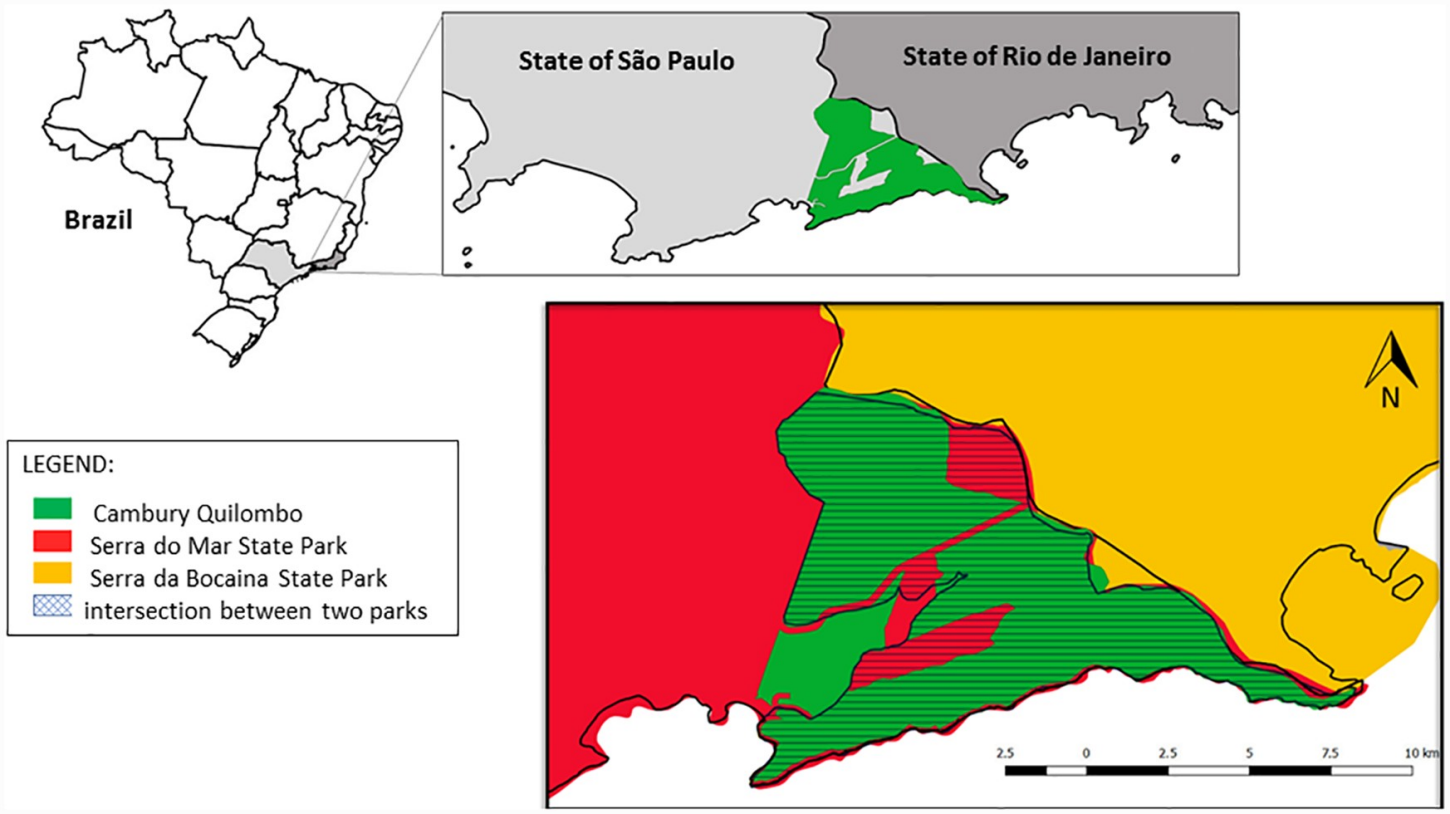
Location of cambury quilombo in Ubatuba, SP, Brazil (Sauini, T).

Bairro do Cambury (BC) [23°21’26.12”S and 44°46’10,46”W], is located in Ubatuba city, São Paulo (Brazil). It is inside the Parque Estadual Serra do Mar (PESM)–Núcleo Picinguaba and Parque Nacional Serra da Bocaina (PNSB), between São Paulo and Rio de Janeiro states. Thus, as a way to characterize the study area, a map of the region where the present study was carried out is presented. One of the authors (Sauini, T) prepared the map (Fig 1) using the free software QGIS (available at: www.qgis.org), using a collection of spatial data from the National Institute of Colonization and Agrarian Reform (available at: http://acervofundiario.incra.gov.br/acervo/acv.php) and the Brazilian Institute of Geography and Statistics (available at: https://mapas.ibge.gov.br/bases-e-referencial/bases-cartograficas/ digital meshes); and using the geographic coordinates reference system "sirgas 200" (Geocentric Reference System for the Americas).

This region remained isolated until 1970 due to its remote location and geography, but the construction of the BR 101 road aided the movement of residents, tourists and real estate brokers, who were attracted by the captivating landscape of the area [20]. People belonging to this traditional community are a mix of indigenous people, Afro-Brazilians and Europeans. According to the ITESP [21], 300 people distributed in 50 families live in this area, there is a school that provides basic education, and the nearest health service is located 25 km from the community. People in urgent medical situations or in need of specific treatments must go to the Hospital Santa Casa de Ubatuba, located 50 km from BC. The predominant religions in this area are Roman Catholicism and evangelicalism.

### Participatory research

During five consecutive meetings held between 2015 and 2016 the desired aims were discussed with representatives of the community, the president of the Quilombo do Cambury Association and some community residents who were interested in participating in this research. It was decided that recording the culture would be conducted through a video and a booklet containing information about their traditional practices, particularly about the plants that are used for different purposes: shipbuilding, handicrafts, medicines. This article focused only on medicinal plants uses.

Fieldwork was conducted over 80 days between September 2016 and January 2018. In the field, three participants, the local partners, were motivated to record their traditional knowledge as local researchers. They participated in all of the steps of research development, from objective definition to decisions made about data recording and analysis and the presentation to the community. To train local partners interested in conducting the research, the researchers from São Paulo Federal University (Universidade Federal de São Paulo) and botanists at the Herbário Municipal (PMSP) offered courses about 1) plant collection and 2) conducting interviews about knowledge of medicinal plants using unstructured interviews [22]. These courses were conducted informally in the community, verbally and demonstratively, for local partners with different levels of education (from illiterate to high school graduates). Since they talk Portuguese, the courses were conducted in this language. Thus, local partners, along with researchers from the university, were responsible for conducting the ethnobotanical survey by selecting and interviewing specialists in medicinal plants, and for collecting the plants. A drying oven was installed in the fieldwork area to dry plants, allowing the community to follow all of the processes of plant identification and deposition.

The selection of interviewees was performed by local partners because they know all of the inhabitants of BC, including medicinal plant specialists. Initially they appointed the older members of the community considering that they would have a comprehensive knowledge of the traditions of the local culture, transmitted by their parents and grandparents. Then, they selected some of the residents for the interviews based on the criterion: “an expert in the use of plants”. According to Johnson [3], participant selection in participatory research is similar to sample selection in a survey—it determines what information is gathered, how robust the results will be and to what extent they can be extrapolated. The interviews were scheduled, and each visit lasted approximately 60 minutes. Furthermore, each interviewee was visited 4 to 6 times. To record local knowledge, of medicinal plants, the local partners conducted unstructured interviews [22]. Personal details (age, sex, ethnicity, source of knowledge) and ethnobotanical data (therapeutic use, part used, formula, recipe, route of administration, dosages, and restrictions on use) were recorded using two forms. University researchers followed local partners during all of the interviews and during the entire process of plant collection, providing any academic or scientific support required. Plants were collected by the drying method [23], botanical data were recorded (habit, type, colour of flower and fruiting) and photographs were obtained, aiding the taxonomical identification of plants. The location of the sample collection was recorded using GPS (g*lobal positioning system*). Data were complemented with participant observations and field diaries [22, 24] by one of the university researchers (co-author TS). With all of the forms completed and the knowledge of the interviewees recorded, the same researcher, along with the local partners, visited the interviewees’s houses to check the data and recollect plants if necessary.

The data obtained were organized as a booklet and a video. During video recordings, the interviewees were asked about the use of plants, beliefs, the presence of the park in this area, the impacts on the community caused by road construction, and the environment and conservation questions, as well as their concerns regarding the heritage that will be left for future generations.

A workshop called “Know-how Exchange” was organized with the participation of the local partners, interviewees, and researchers. This meeting was important for checking, for the last time, the obtained data and discussing the format of the booklet and the video. To create a final version, researchers from the university brought booklets previously published by other authors during other projects, as a starting point for deciding which content to add to their booklet. The most commonly used plants in the community were selected by the interviewees to be published in the booklet.

## Results and discussion

### Participatory research

Three community members womans with age that ranged enter 40 e 55, and resident in the area for more 20 years. These participated (co-authors CC, MAS and MASG) individuals who showed interest in conducting the ethnobotanical study during the first meetings held by the project's coordination in the community. They played an active fundamental role in the development of this work in all of the steps, from the objective definition to the recording, analysis and publication of the data. According to Grasser et al. [25], an ethnobiological survey should allow for the participation of the community at all steps and provide access to all of the collected data in order to bring benefits to the local communities.

Paniagua-Zambrana et al. [26] showed that a combination of participants and interviewers from the local community itself could be an interesting strategy for obtaining data in ethnobotanical studies. Based on the collaboration of the local participant, in addition to being a facilitator and / or mediator in the fields, it can make it possible to collect data that are more contextualized with local perceptions.

However, in some cases local partners may have difficulties in some research sensitive data and/or they could have relationships problems. This fact can bring fragility to the data and therefore integration with external researchers can facilitate in this case.

It is important to integrate the knowledge of local partners with academic researchers, since it values and records their results from a local perspective (from within the community) that is, the emphasis is on the emic approach [27], in complementarity with local knowledge and academic. In this way, it also reinforces research as having an ethical approach, according to recent studies focusing on collaborative research that seeks to tell the history of biodiversity and its potential for use, not only to catalogue species [28; 29].

In this sense, it was important to consider the following points: 1) This study was performed based on the field efforts of residents (local partners) and researchers from the university, and the residents were conducting an academic study for the first time; 2) The data collected were the results of interviews with nine residents living in BC (qualitative research) with the local partners choosing who and how many would be interviewed. 3) During the study, training for the collection of ethnobotanical data was offered to local partners, to align the methodologies to be performed in the data collection. Such activities counted as research time, an unusual fact in ethnobotanical and / or ethnopharmacological research. 4) For the local partners involved in the study—“time” was different from that of urban society, a fact that caused difficulties throughout the absorption of concepts during the ethnobotanical study and in data collection. 5) Local partners perform other daily activities that guarantee their livelihoods and must reconcile these activities with fieldwork. Therefore, the data obtained here are very robust and integrated with local perceptions, compared to other ethnobotanical studies developed in traditional communities of the Atlantic Forest [17, 30, 31], Amazonian forest [32, 33], scrublands of the Brazilian Cerrado [34], and Pantanal wetlands [35]. In this sense, 82 medicinal plants were registered in this study, and 148 other plants with different ethnobotanical uses, such as: food, shipbuilding, construction, combustion, dyes and handicrafts, were registered by residents in this same survey but the data are actually unpublished.

### Local partners

During this work, at this deeper level of participatory research, there were some limitations and facilities, according to the expectations of the university researchers and the routines and the way of life of each participant in the community. Initially, for example, a small group of people were interested in participating, but few of them actually involved. Among these people, there was a difficulty in relation to the time required to perform the research since as each one had their daily routine and tasks, in addition to caring for their families, business and farms, in order to obtain daily living. Another difficulty was in relation to communication with the interviewees, who at the beginning of the field work had no internet or telephone signal in the community. Thus, the work was often hampered due to the unavailability of the interviewees.

Three of the local partners were female and were 39–50 years old. One of them had been born in BC, and the other two had been born in the Ubatuba region; they had moved to BC after marrying local residents. They are an artisan, a cook and a merchant, and none of them completed their high school education. One of them did not know to write and needed help from the university researchers during the recording of traditional knowledge.

All of them believe that this record is important “*to guarantee that the knowledge and traditional practices of the culture are not lost over the years*, *as many others have already been lost*”, as explained by one of the interviewees. The local partners were responsible for explaining to the interviewees the project and the importance of recording traditional knowledge for the residents about the plants, as discussed in the community meetings that preceded the beginning of the fieldwork.

### Specialists in medicinal plants

Among the nine interviewees with specialized knowledge of medicinal plants, seven were male (77.8%), and two were female (22.2%); they were between 35 and 50 years old, and four of them were older than 50. All of them had incomplete fundamental educations (without completing high school), among them were a fisherman, a cook, farmers, a bricklayer and a craftsman.

Most of them (five) had been born in BC and had parents and relatives that were also born there. The main route of knowledge acquisition was oral because they lived together with ancestors who used natural resources as daily practices, as observed in other traditional communities [36, 37].

Concerning religiosity, a strong influence of evangelical Christianity on the local culture was observed, and medicinal practitioners and/or symbolic aspects related to the Catholic religion and/or Afro-Brazilian religions, which are common in Brazilian cultures, were not found. These findings have been observed in different communities in Brazil. Zank and Hanazaki [38] observed a diminished number of healers in the Quilombola area of Santa Catarina state (in the municipalities of Garopaba—Morro do Fortunato and Aldeia communities and the Paulo Lopes—Santa Cruz community). The spread of evangelism could have some impact on traditional treatment because this religion has certain taboos about the traditional use of plants and religious practices performed by Afro-Brazilian and indigenous communities [39]. In ethnic studies conducted by Rodrigues [32] and Santos et al. [33] among Amazonian River dwellers in the Tapiira community, Unini River, 10 healers (including midwives, prayers and mediums) with Catholic and Afro-Brazilian influences were recorded in this area in 1995, and all of the healers used prayers during healing practices. In contrast to the findings reported in 1995, studies conducted by the same team in 2012 showed no healers in the community because the local religion had been changed by evangelism (data not published).

The dynamics of religious change over time in some places are comprehensible; however, they cause concerns when they affect and compromise the collective local health, as is occurring in the Amazon, where access to conventional medicine is limited.

Despite the transformation that the community is undergoing in BC, some characteristics of traditional practices persist, such as the tradition of collecting plants during a specific phase of the moon to avoid plant parasites and to not compromise their use. The influence of the moon was also observed and discussed in other studies; for example, Jovchelevich and Camara [40] wrote about the moon phases and their influence on carrot (*Daucus carrotta*) yields. The study showed variations in the new and full phases, and the results obtained were better when sowing occurred during the new moon.

### The medicines: Recipes and therapeutic uses

**Fig 2:**
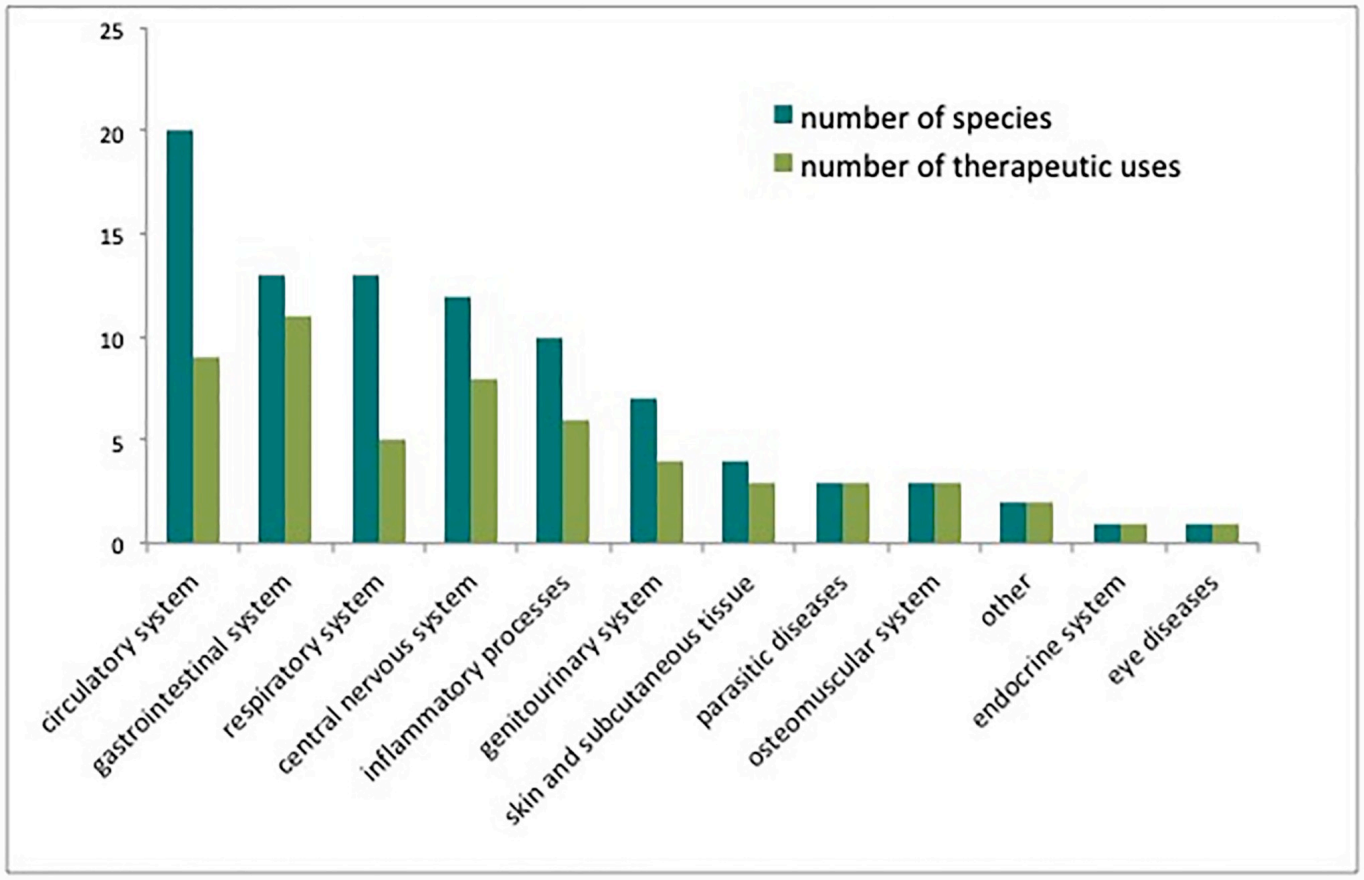
Total number of medicinal plant species indicated for each of the 12 categories of medicinal uses.

Eighty-two species of plants were indicated for 55 therapeutic uses, and 90 recipes (Table 1). They were grouped, with the aid of local partners, into 12 categories of medicinal use: circulatory system (20 species and 9 uses), gastrointestinal system (13 and 11), respiratory system (13 and 5), central nervous system (12 and 8), inflammatory process (10 and 6), genitourinary system (7 and 4), skin diseases (4 and 3), parasitic diseases (4 and 2), osteomuscular system (3 and 3), other uses (2 and 2), endocrine system (1 and 1) and the ocular system (1 and 1) (as seen in Fig 2 and Table 2). Previous studies have also found that the two first categories, which had the largest numbers of uses above, were the most frequent in other communities, such as the Atlantic forest community [31, 41–44]. The large number of plant species indicated for gastrointestinal disorders in these studies could be associated with the absence of basic sanitation, which can account for a variety of diseases linked to the faecal-oral route, since the majority of the therapeutic uses of this category refer to the treatment of stomach ache. The samples were identified by botanists according to the nomenclature of the site “Flora do Brasil 2020 (under construction” and available at: www.floradobrasil.jbrj.gov.br) for native Brazilian species and by the site “The Plant List” (available at: www.theplantlist.org), for exotic species.

**Table 1:** The 82 medicinal species registered by the local partners during the ethnobotanical survey in the “Bairro do Cambury”. The same species may belong to more than one of the 12 categories of use.

Family	Species (voucher)	Vernacular name(s) in Portuguese	Therapeutic use (emic—etic terms)	Part	Recipe	Routes
**CATEGORY: CIRCULATORY SYSTEM– 20 species**						
Acanthaceae	*Hypoestes phyllostachya* Baker–MAS076	ferromicina	*machucadura (*hematoma)—haematoma	le	de	or
Alismataceae	*Echinodorus grandiflorus* (Cham. & Schltdl.) Micheli*–CC030	chapéu-de-couro	hematoma—haematoma	le	de	tr
Amaranthaceae	*Alternanthera brasiliana* (L.) Kuntze*–THS083	ferramicina	*machucadura—*haematoma	le	ma	tr
	*Dysphania ambrosioides* (L.) Mosyakin & Clemants–THS191	erva-santa-maria	*Cortes*, *feridas e machucadiura* (hematoma)—coagulant, wounds and haematoma	le	ma	tr
Araceae	*Xanthosoma taioba* E.G.Gonç.*–MA089	taioba	*anemia—*anaemia	le	de	or
Asteraceae	*Ageratum conyzoides* L.*–MA093	erva-são-joão	*limpar o sangue—*depurative	le	de	or
	*Erechtites valerianifolius* (Wolf) DC.*–MA040	capirosoba	*anemia e engrossar o sangue—*anaemia	le	de	or
Bignoniaceae	*Handroanthus albus* (Cham.) Mattos*–PBY090	ipê-amarelo	*bom pro sangue—*depurative	ba	de	or
Convolvulaceae	*Ipomoea batatas* (L.) Lam.–MA088	batata-doce	*anemia—*anaemia	le	co	or
Fabaceae	*Cajanus cajan* (L.) Huth–THS072	feijão-guandú	*anemia—*anaemia	le	de	or
	*Swartzia* cf. *oblata* R.S.Cowan*–MA019	jatobá	*engrossar o sangue—*anaemia	le	de	or
	*Hymenaea* cf. *altissima* Ducke*–THS132	jatobá	*para o sangue—*depurative	le	de	or
Lythraceae	*Cuphea carthagenensis* (Jacq.) J.F.Macbr.*–CC016	sete-sangria	*acalma pressão alta—*antihypertensive	ro	de	or
Meliaceae	*Cabralea canjerana* (Vell.) Mart.*–MAS052	ingá-cajarana	*bom pro sangue—*depurative	ba	de	or
Rutaceae	*Citrus* x *limon* (L.) Osbeck ◆ –GDS040	limão	*afina o sangue—*anticoagulant	fr	ma	or
Solanaceae	*Solanum scuticum* M.Nee*–MAS030	jurubeba	*para o sangue—*depurative	ex	na	or
Urticaceae	*Urera nitida* (Vell.) P.Brack*–MA095	urtiga	*boa pro sangue—*depurative	le	na	or
Verbenaceae	*Stachytarpheta cayennensis* (Rich.) Vahl*–THS168	gervão	*anemia—*anaemia	le	de	or
Zingiberaceae	*Alpinia zerumbet* (Pers.) B.L.Burtt & R.M.Sm.–THS166	colônia	*machucadura—*haematoma	fr	ab	tr
	*Renealmia petasites* Gagnep.*–THS030	pacová	*anemia—*anaemia	ro	de	or
**CATEGORY: GASTROINTESTINAL SYSTEM– 13 species**						
Apiaceae	*Centella asiatica* (L.) Urb.–MA091	centella -asiática	*dor de barriga—*diarrhea	le	de	or
	*Eryngium foetidum* L.*–CC009	coentro	*digestão—*digestive	le	na	or
Asteraceae	*Baccharis dracunculifolia* DC.*–THS070	alecrim	*digestão—*digestive	le	na	or
	*Baccharis* L. Sect. Caulopterae DC.–PBY074	carqueja	*dores no estômago—*stomachache	le	de	or
	*Bidens pilosa* L.–CC019	picão	*hepatite—*hepatitis	le	de	or
	*Gymnanthemum amygdalinum* (Delile) Sch.Bip. ex Walp.–MA009	estomalina	*Ressaca*, *fígado—*hangover recovery	le	de	or
	*Tithonia diversifolia* (Hemsl.) A.Gray–MA014	margaridão	*gastrite*, *úlcera e ressaca—*gastritis, ulcer and hangover recovery	le	de	or
Cannabaceae	*Trema micrantha* (L.) Blume*–THS157	gandiúba	*hemorróida—*hemorrhoid	ro	de	vr
Clusiaceae	*Garcinia gardneriana* (Planch. & Triana) Zappi*–THS153	bacubari	*hepatite—*hepatitis	ro	de	or
Myrtaceae	*Eugenia uniflora* L.*–CC007	pitanga	*enjôo e estômago—*stomachache	le	de	or
	*Psidium guajava* L. ◆- MA106	goiaba	*diarreia—*diarrhea	le	de	or
Poaceae	*Imperata* sp.–CC030	sapê	*Evita diarreia (quando os dentes das crianças estão nascendo)—*diarrhea	le	de	or
Rutaceae	*Zanthoxylum rhoifolium* Lam.*–*THS028	mamica	*hepatite*	le	de	or
**CATEGORY: RESPIRATORY SYSTEM– 13 species**						
Asteraceae	*Elephantopus mollis* Kunth*–MA077	algodão	*para o pulmão—*to the lung	le	ma	or
	*Mikania* cf. *glomerata* Spreng.*–MAS062	salomão-de-gambá	*antigripal—*flu	le	co	or
	*Mikania laevigata* Sch.Bip. ex Baker*–CC026	caroba-branca	*tosse*—antitussive	le	sy	or
	*Vernonanthura beyrichii* (Less.) H.Rob.*–MA011	cajuna	*pneumonia e tosse—*pneumonia and antitussive	le	ma	or
Boraginaceae	*Varronia curassavica* Jacq.*–THS173	baleeira	*pulmão—*to the lung	le	de	or
Crassulaceae	*Kalanchoe crenata* (Andrews) Haw.–CC002	saião	*pneumonia—*pneumonia	le	de	or
Lamiaceae	*Mentha pulegium* L.–PBY107	poejo	*tosse—*antitussive	le	sy	or
Musaceae	*Musa* sp.–PBY069	bananeira	*pneumonia e tosse—*pneumonia and antitussive	cl	ma	or
Myrtaceae	*Plinia edulis* (Vell.) Sobral*** –MA100	cambucá	*gripe—*flu	le	de	or
Piperaceae	*Piper mollicomum* Kunth** –MAS072	jaburandi	*pulmão*—to the lung	le	de	or
Pteridaceae	*Adiantum raddianum* C. Presl*–MAS074	avenca	*pulmão—*to the lung	le	ma	or
Urticaceae	*Cecropia glaziovii* Snethl.*–THS178	embaúba	*bronquite—*bronchitis	le	sy	or
	*Cecropia pachystachya* Trécul*–MA042	embaúba-branca	*tosse—*antitussive	le	sy	or
**CATEGORY: CENTRAL NERVOUS SYSTEM– 12 species**						
Amaranthaceae	*Pfaffia glomerata* (Spreng.) Pedersen*–CC15	novalgina-de-gominho	*febre—*fever	le	de	or
Apiaceae	*Foeniculum vulgare* Mill.–CC025	erva-doce	*calmante—*anxiolytic	le	de	or
Asteraceae	*Achillea millefolium* L.*–*CC020	novalgina	*dores—*pain	le	de	or
	*Achyrocline flaccida* (Weinm.) DC.*–MA041	marcela	*dor de cabeça e calmante—*headache and anxiolytic	cl	de	or
Bignoniaceae	*Cybistax antisyphilitica* (Mart.) Mart.*–MAS028	cinco-folhas	*dor nas costas e corpo*—back and body aches	ba	de	or
Fabaceae	*Andira fraxinifolia* Benth.*–MA102	sucupira	*dor no joelho—*knee pain	le	ma	tr
Lamiaceae	*Ocimum campechianum* Mill.*–CC010	favaca	*dor no corpo—*body aches	le	de	or
	*Ocimum gratissimum* L.–CC014	favacão	*dores no corpo—*body aches	le	de	or
Piperaceae	*Piper scutifolium* Yunck** –MAS086	jaborandi	*anestésico bucal—*oral anesthetic	ro	na	or
Poaceae	*Cymbopogon citratus* (DC.) Stapf–CC023	capim-limão (cidreira)	*calmante—*anxiolytic	le	de	or
Polygalaceae	*Polygala paniculata* L.*–MA111	gelol / alecrim-do -campo	*dores—*pain	wp	ab	tr
Verbenaceae	*Lippia alba* (Mill.) N.E.Br. ex P.Wilson*–SB016	melissa	*calmante—*anxiolytic	le	de	or
**CATEGORY: INFLAMATORY PROCESS– 10 species**						
Acanthaceae	*Hypoestes phyllostachya* Baker–MAS076	ferromicina	*tirar dor e inflamação—*anti-inflammatory	le	de	or
Asteraceae	*Cyrtocymura scorpioides* (Lam.) H.Rob.*–MA082	mata-pasto	*furúnculo—*furuncle	le	ma	tr
Moraceae	*Morus nigra* L.–GDS068	amora	*anti-inflamatório—*anti-inflammatory	le	de	or
Myrtaceae	*Myrcia spectabilis* DC.*–THS060	arueira	*inflamação na garganta—*sore throat	ba	de	or
	*Plinia* sp.–MA101	jaboticaba	*anti-inflamatório—*anti-inflammatory	fr	na	or
Plantaginaceae	*Plantago australis* Lam.*–MA044	tansagem	*dor de garganta—*sore throat	le	de	or
Poaceae	*Imperata* sp.*–*CC030	sapê	*quando os dentes das crianças estão nascendo—*toothache when the children's teeth are being born	le	de	or
Rutaceae	*Dictyoloma vandellianum* A.Juss.*–MA130	carobinha	*anti-inflamatório—*anti-inflammatory	le	de	tr
Solanaceae	*Solanum americanum* Mill.*–SB023	erva-moura	*problemas "por dentro" e infecção—*problems "inside" and infection	le	de	or
	*Solanum capsicoides* All.*–MA128	rebenta-cavalo	*furúnculo (soltar o pus)—*furuncle	fr	ra	tr
**CATEGORY: GENITOURINARY SYSTEM– 7 species**						
Asteraceae	*Chaptalia nutans* (L.) Pol.*–MA133	dente-de-leão	*rim—*kidney stone	wp	de	or
	*Erechtites valerianifolius* (Wolf) DC.*–MA040	capirosoba	*cólica—*menstrual cramps	le	de	or
Costaceae	*Costus arabicus* L.*–MA119	caninha	*para o rim—*kidney stone	le	na	or
	*Costus spiralis* (Jacq.) Roscoe*–THS079	caninha-do-brejo	*para o rim—*kidney stone	le	na	or
Fabaceae	*Swartzia oblata* R.S.Cowan*–MA019	jatobá	*útero—*myoma	cl	de	or
Lauraceae	*Persea americana* Mill. ◆– GDS004	cinco-folhas	*pedra no rim—*kidney stone	le	co	or
Phyllanthaceae	*Phyllanthus niruri* L.*–MA075	erva-pedra	*para o rim—*kidney stone	le	de	or
**CATEGORY: SKYN AND SUBCUTANEOUS TISSUE DISEASES– 4 species**						
Asteraceae	*Cyrtocymura* cf. *scorpioides* (Lam.) H.Rob.*–MA082	maria-pretinha	*frieiras—*antimycotic	le	ma	tr
	*Conyza* sp.1 –MA079	rabo-de-cavalo	*pano branco—*antimycotic	le	ma	tr
Malvaceae	*Gossypium* sp.–SB029	algodão	*para amarrar o umbiro*, *após o parto*- to tie the navel after birth	fr	to tie	tr
Siparunaceae	*Siparuna brasiliensis* (Spreng.) A.DC.*–MA136	salomão-de-gambá	*frieiras—antimycotic*	le	de	tr
**CATEGORY: PARASITIC DIASEASES– 4 species**						
Asparagaceae	*Furcraea* cf. *foetida* (L.) Haw. ◆ –MAS033	pita	*sarna—*scabies	le	na	tr
Bignoniaceae	*Jacaranda puberula* Cham.**–MA056	caroba-branca	*sarna—*scabies	le	ab	or
Lamiaceae	*Aegiphila integrifolia* (Jacq.) Moldenke*–MAS036	cajuna	*sarna—*scabies	le	de	tr
Thelypteridaceae	*Macrothelypteris torresiana* (Gaudich.) Ching*–MA092	samanbaiazinha (feno grego)	*verme—*helminthiases	le	ma	or
**CATEGORY: OSTEOMUSCULAR SYSTEM– 3 species**						
Bignoniaceae	*Cybistax antisyphilitica* (Mart.) Mart.*–MAS028	cinco-folhas	*dor nas costas—*back pain	ba	de	or
Crassulaceae	*Kalanchoe crenata* (Andrews) Haw.–CC024	saião	*colar osso—*fractures	wp	ab	tr
Fabaceae	*Andira fraxinifolia* Benth.*–MA102	sucupira	*dor no joelho—*knee pain	le	ma	tr
**CATEGORY: ENDOCRINE SYSTEM– 1 specie**						
Cucurbitaceae	*Cayaponia* cf. *tayuya* (Vell.) Cogn.*–MA126	taiuiá	*diabetes—*diabetes	cl	ab	or
**CATEGORY: EYE DYSEASES– 1 specie**						
Dilleniaceae	*Davilla rugosa* Poir.*–MA035	cipó-caboclo	*irritação nos olhos ou cegueira de animais—*eye irritation or animal blindness	ex	na	tr
**CATEGORY: OTHER– 2 species**						
Poaceae	*Cymbopogon nardus* (L.) Rendle–MA025	citronela	*repelente—*mosquito repellent	le	ab	tr
Solanaceae	*Solanum sessiliflorum* Dunal*–THS039	cubuí	*câncer—*cancer	fr	na	or

Alcoholic beverage–ab; Bark—ba; Bulb–bu; Cook–co; Decoction–de; Exudate–ex; Flower—fl; Fruit—fr; Infusion–in; Heat–he; Inhalatory Route—ir; In natura—na; Leaves—le; Maceration–ma; Oftalmic Route–ofr; Oral Route–or; Otologic Route–otr; Roast–ra; Root–ro; Seeds–se; Syrup–sy; Transdermic Route–tr; Vaginal Route–vr; Whole plant–wp.

native to Atlantic forest (according to Flora 2020 [45]);

** native and considered to be endangered or vulnerable according to the ICMBio (Instituto Chico Mendes Biodiversity Conservation) database [46];

*** considered threatened according to the Brazilian Flora Red Book [47].

^◆^ exotic and invasive.

**Table 2:** Number and percentage of therapeutic uses (emic and etic terms) and of species belonging to the categories of medicinal uses indicated by the 9 interviewees from “Bairro do Cambury”.

Categories of medicinal uses	Number and (%) of therapeutic uses	Number and (%) of species
1. Circulatory System	*Machucadura—*haematoma	*20 (22*,*2%)*
	*Feridas na pele—*wounds	
	*Cortes—*coagulant	
	*Anemia—*anaemia	
	*Limpar o sangue—*depurative	
	*Engrossar o sangue—*anaemia	
	*Bom pro sangue—*depurative	
	*Acalma pressão alta—*antihypertensive	
	*Afina o sangue—*anticoagulant	
	*9 (16*,*4%)*	
2. Gastrointestinal System	*Dor de barriga—*diarrhea	13 (14,4%)
	*Digestão—*digestive	
	*Dores no estômago—*stomachache	
	*Hepatite—*hepatitis	
	*Ressaca/fígado—*hangover recovery	
	*Gastrite-* gastritis	
	*Úlcera—*ulcer	
	*Hemorroida—*hemorrhoid	
	*Diarreia—*diarrhea	
	*Enjôo—*stomachache	
	*Evita diarreia—*diarrhea	
	*11 (20%)*	
3. Respiratory System	*Para o pulmão—*to the lung	13 (14,4%)
	*Antigripal—*flu	
	*Tosse—*antitussive	
	*Pneumonia—*pneumonia	
	*Bronquite—*bronchitis	
	*5 (9*,*1%)*	
4. Central Nervous System	*Febre—*fever	12 (13,3%)
	*Calmante—*anxiolytic	
	*Dor de cabeça—*headache	
	*Dores—*pain	
	*Dor nas costas–*back pain	
	*Dor no corpo—*body aches	
	*Dor no joelho—*knee pain	
	*Anestésico bucal—*oral anesthetic	
	*8 (14*,*5%)*	
5. Inflammatory Processes	*Inflamação—*anti-inflammatory	10 (11,1%)
	*Furúnculo—*furuncle	
	*Dor de garganta—*sore throat	
	*Problemas “por dentro”—*problems "inside"	
	*Infecção—*Infection	
	*Quando os dentes de criança nascem—*toothache when the children's teeth are being born	
	*6 (11%)*	
6. Genitourinary System	*Cólica—*menstrual cramps	7 (7,8%)
	*Rim—*kidney stone	
	*Útero—*myoma	
	*Pedra no rim—*kidney stone	
	*4 (7*,*3%)*	
7. Skin and Subcutaneous Tissue Diseases	*Frieiras—antimycotic*	4 (4,4%)
	*Pano branco* (micose)—antimycotic	
	*Para amarrar umbigo após o parto—*to tie the navel after birth	
	*3 (5*,*5%)*	
8. Parasitic Diseases	*Sarna—*scabies	4 (4,4%)
	*Verme—*helminthiases	
	*2 (3*,*6%)*	
9. Osteomuscular System	*Dor nas costas—*back pain	3 (3,3%)
	*Colar osso—*fractures	
	*Dor no joelho—*knee pain	
	*3 (5*,*5%)*	
10. Other	*Repelente—*mosquito repellent	2 (2,2%)
	*Câncer—*cancer	
	*2 (3*,*6%)*	
11. Endocrine System	*Diabetes—*diabetes	1 (1,1%)
	*1 (1*,*8%)*	
12. Eye Diseases	*Irritação nos olhos ou cegueira de animais—*eye irritation or animal blindness	1 (1,1%)
	*1 (1*,*8%)*	
**Total (%)**	**55 (100%)**	**90 (100%)**

The indicated medicinal species total 82; however, as the same species may belong to more than one category of medicinal use, they total 90 species, according to the table above.

Other studies developed in traditional communities of the Atlantic forest have also recorded large numbers of medicinal plants directed for treating respiratory system diseases [31, 43], and according to Almeida and Albuquerque [48], this outcome occurred because respiratory diseases are more common in this biome and because their symptoms are clearer, thus favouring the identifications of these diseases and the identification of these plants.

Fifty-four species (65.8%), indicated with the asterisk in Table 1, are native to the Atlantic forest, while the others are exotic or naturalized or have not been fully identified, demonstrating the floral diversity of the region, which was influenced by European and African people during the civilizing process during the colonial period in Brazil. Some ethnobotanical/ ethnopharmacological studies developed in Brazil have shown a higher representation of native species among Afro-Brazilians living in the Pantanal wetlands [35], among the 31 species indicated, 25 were native. Among Indians living in the Cerrado [34], all 138 indicated species were native to the biome, and among river dwellers, 66% of 120 recorded species were native to the Amazon forest [32]. In contrast other studies developed in Brazil have shown a higher representation of non-native species among traditional communities living in the Atlantic forest [17, 49–52], in the Amazon forest [53] and in Caatinga [54]. Thus, there are no patterns to the plants used in traditional medical systems, independent of the biome or the human group. Regarding the Atlantic forest, one possible reason for this lack of a pattern is that plants from Europe, Africa and Asia were introduced and popularized in traditional communities of this biome since colonial times, along with the cultural changes and ethnic miscegenation that occurred over five centuries [49].

The four species marked with ** in Table 1 are native to the Atlantic forest and considered to be endangered or vulnerable according to the ICMBio (Instituto Chico Mendes Biodiversity Conservation) database [46], and of these, the one, marked with ***, is considered threatened according to the Brazilian Flora Red Book [47]. In addition, the four marked with ◆ are exotic and invasive.

In Table 1, therapeutic uses are presented in italics and in the Portuguese language according to emic terms as indicated by the interviewees, as well as by their respective etic terms, for example: *machucadura*—haematoma. This community has access to conventional medicine in hospitals in the municipalities of Ubatuba and Paraty, located 49,5 and 29 km away, respectively, indicating that conventional medicine is commonplace in the daily lives of residents. When a therapeutic use referring to any of the indicated plants was presented with an emic term by the interviewees, the university researchers consulted the local partners and correlated the emic and ethical terms accordingly. Participant observations and field diaries [22, 24] were recorded by the co-author (TS) to capture the perceptions of interviewees and local partners regarding their relationships with plants, emic terms for the plants, and their explanations and other observations that were pertinent to understanding those relationships. These methods and techniques sought to complement the quality of the data collected by the local partners.

In the column “Therapeutic Use”, two types of information are observed: information concerning the possible activities of plants (digestive, for example) and information concerning the symptoms and/or diseases that they treat (hepatitis, for example). Because this study used terms for the categorization of medicinal use and they were indicated by the interviewees, we decided to keep them in Table 1 to diminish the chance of misinterpretation.

The majority of interviewees gave names to species with the same popular name and used them for the same therapeutic purposes, such as *sucupira* (*Andira franxinifolia* Benth) which was indicated by two interviewees for knee trouble, and the traditional recipe was the same. However, there were two cases in which the same plant was indicated for two different conditions: *saião* [*Kalanchoe crenata* (Andrews) Haw.] indicated to heal bone fractures and pneumonia, and ferromicina (*Hypoestes phyllostachya* Baker), indicated for haematoma and for pain relief and inflammation.

These 90 recipes were prepared as teas via infusion or decoction or as alcoholic beverages, syrups, and applications, via chewing and maceration, with decoction (56.7%) being the most commonly used method, followed by maceration (14.5%). Ingestion was the most cited route of administration (77.8%). Ethnobotanical studies conducted by Silva et al. [30] and Tuler [55] found that decoction was the most common method of preparation for medicinal plants. This finding was also observed in other studies, which also suggested that decoction and infusion are used because the methods are fast, easily accessible and inexpensive [44, 56, 57].

Different parts of plants were used in recipes however, leaves were the most commonly used part of plants employed in medicinal recipes (72.2%), followed by fruits (6.7%), among others. The predominance of the use of leaves can be related to the relative ease of obtaining them; their use was verified in other studies and is considered an important way to collect plant material since harvesting leaves is less harmful to the plant than harvesting other plant material [30, 29, 36, 55].

In most of the recipes, a single plant was part of the recipe, with or without other complements, such as salt, sugar, honey, lemon, alcohol, vinegar, milk and even animal parts. Sugar was used to prepare syrups, such as in the recipe for *poejo* (*Mentha pulegium* L.) or as sweeteners of drinks and teas (prepared by infusion). Honey was considered the best way to sweeten medicines because it is considered “natural” and less aggressive to the organism, in addition to being found in the backyards of the residents. Lemon, honey and *guaco* (*Mikania* cf. *glomerata* Spreng.) were used in recipes to treat flu. Alcohol was usually used to prepare alcoholic beverages in the form of “cachaça” (an alcoholic beverage). Milk was used as one complement for softening the taste of some plants, such as *saião* [*Kalanchoe crenata* (Andrews) Haw.], which is indicated to treat pneumonia. There were few recipes with more than one plant (poly-recipes), such as the following: leaves of *pitanga* (*Eugenia uniflora* L.) and leaves of *goiaba* (*Psidium guajava* L.) to treat nausea and stomach troubles and the leaves of *cajuna [Aegiphila integrifolia* (Jacq.) Moldenke], *caroba* (*Jacaranda puberula* Cham.) and *cinco-folhas* [*Cybistax antisyphilitica* (Mart.) Mart.] to treat scabies.

Finally, parts of animals were indicated in some recipes, as in the case of a recipe to treat athlete's foot that employs chicken fat and *maria-pretinha* (*Cyrtocymura scorpioides* (Lam.) H.Rob). The other 12 animal parts were either associated or not with medicinal plants in recipes; a complete list will be available in a future publication.

The concomitant use of synthetic drugs and medicinal plants was not observed. The interviewees stated that it is not necessary to obtain medicaments in drug stores because they have all of the medicines that they need for daily use in their backyards. It is also important to emphasize the distance between the community and the closest cities (Ubatuba and Paraty), the difficulties related to transport from BC to urban centres, and the price of synthetic drugs. Conversely it has been considered easy to cultivate the cited plants and consequently to yield the drugs derived from these medicinal plants, which in most cases, have superior effects compared with their synthetic counterparts, according to the interviewees.

In this community, it was observed that biomedicine complements traditional medicine only when the disease is more aggressive, forcing the resident to go to the hospital.

### Data records: Video and booklet

During the interviews photographic and video recordings were obtained of the interviewees, and plant collection and other fieldwork was conducted. A video was produced (Quilombola Heritage–available in YouTube, with subtitles in Portuguese and English, (https://www.youtube.com/watch?v=yxpOWv3u6RQ&t=2524s).

The booklet entitled “Quilombo do Cambury, Saberes e Tradições” (Cambury Quilombo, Knowledge and Traditions) contain information about medicinal and non-medicinal plants used in the community (for handicrafts, dyes, construction, etc.); in addition, it illustrates the opinion of residents about the importance of this record and conservation of the environment to future generations. The booklet is available to download (https://issuu.com/thasauini/docs/livreto_cambury_thamara_final_13.06). In the booklet 10 species of medicinal plants were included because they are more relevant to the community according to the interviewees and local partners. Information regarding the possibly toxic effects of these plants was added if it was found in the scientific literature, thus promoting the union between science and community knowledge. In addition, other relevant species used in ethnobotany categories (food/seasoning, handicrafts, construction, among others) were included, which will be the subject of another paper.

In addition to virtual versions of the video and booklet, physical copies of each were delivered to the community, where they are freely available to all members of the community, allowing the knowledge to be shared with the successors, helping to reinforce the importance of the knowledge of the community members and the roles of local researchers.

## Conclusions

The present survey is a concrete example of a participatory approach in ethnobotany/ethnopharmacology, in which the active participation of local residents recording their culture enhances their empowerment in decision-making regarding the use of the resources available in their environment.

Even with some limits observed, local employees met the expectations for participation and were present from the definition of the objectives to the dissemination of data to the community itself.

The development of this study contributes to advances in ethnobotany/ethnopharmacology methods, promoting the participation of residents and the recording of their knowledge. This methodology proved to be indispensable during this research, and showed a greater degree of involvement when comparing to many others ethnobotanical studies, since the community members themselves participated actively in all stages of its development. Therefore, this study shows that it is possible to train local inhabitants to record their own knowledge, which in turn can be a valuable tool for protecting the traditional knowledge of different cultures, since current discussions related to intellectual property is a global concern.

## References

[1] Goebel A. Process, perception and power: notes from participatory research in a Zimbabwean resettlement area. Development and Change—The Hague Then London. 1998; 29: 277–305.

[2] Mosse D. People’s knowledge, participation and patronage: operations and representations in rural development In: Cooke B., Kothari U. (Eds.). Participation: The New Tyranny? New York: Zed Books; 2001 p. 16–35.

[3] Johnson N, Lilja N, Ashby JA, Garcia JA. The practice of participatory research and gender analysis in natural resource management. Natural Resources Forum. UK- USA. 2004; 28: 189–200.

[4] Etkin NL, Ticktin T. Integrating Ethnographic and Ecological Perspectives for Ethnopharmacology Field Research. 2005; 1.

[5] Ericson J. A participatory approach to conservation in the Calak- mul Biosphere Reserve, Campeche, Mexico. Landsc Urban Plan. 2006; 74, 242–266.

[6] Gilmore MP, Young JC. The use of participatory mapping in ethnobiological research, biocultural conservation, and community empowerment: a case study from the Peruvian Amazon. J Ethnobiol. 2012; 32: 6–29.

[7] Medley KE, Kalibo HW. An ecological framework for participatory ethnobotanical research at Mt. Kasigau, Kenya. Field Method. 2005; 17: 302–314.

[8] Kalibo HWM, Kimberly E. Participatory resource mapping for adaptive collaborative management at Mt. Kasigau, Kenya. Landsc Urban Plan. 2007; 82: 145–158.

[9] Hitziger M, Heinrich M, Edwards P, Poll E, Lopez M, Krutli P. Maya phytomedicine in Guatemala, Can cooperative Research change ethnopharmacological paradigms? J Ethnopharmacol. 2016; 186: 61–72. 10.1016/j.jep.2016.03.040 27013096

[10] Yazbek PB, Matta P, Passero LF, Dos Santos G, Bragad S, Assunção L et al Plants utilized as medicines by residents of Quilombo da Fazenda, Núcleo Picinguaba, Ubatuba, São Paulo, Brazil: A participatory survey. J Ethnopharmacol. 2019, 244, 112–123. 10.1016/j.jep.2019.112123 31356967

[11] Schultes RE, Von Reis S. Ethnobotany: Evolution of a Discipline. Timber Press, Portland, Oregon; 2008.

[12] Cunninghan AB. Applied Ethnobotany: People, Wild Plant Use and Conservation. In: Conservation Manual. Earthscan Publications Ltd. London; 2001.

[13] IBGE–Instituto Brasileiro de Geografia e Estatística, 2018. https://ww2.ibge.gov.br/home/presidencia/noticias/21052004biomashtml.shtm. Accesed 16 december 2018.

[14] CEPF. Critical Ecosystem Paternship Fund [web page]. 2018. https://www.cepf.net/node/1996. Accessed on November 26, 2018.

[15] Fundação SOS Mata Atlântica e Instituto Nacional de Pesquisas Espaciais (INPE). Atlas dos Remanescentes Florestais da Mata Atlântica—Mapeamento dos Sistemas Coseiros. 2018. http://mapas.sosma.org.br/site_media/download/SOSMA_Atlas-da-Costa_Final.pdf. Accessed 17 july 2018.

[16] Barroso RM, REIS A, Hanazaki N. Etnoecologia e etnobotânica da palmeira juçara (Euterpe edulis Martius) em comunidades quilombolas do Vale do Ribeira, São Paulo. Acta Bot. Bras. 2010; 24: 518–528.

[17] Garcia D, Domingues MV, Rodrigues E. Ethnopharmacological survey among migrants living in the Southeast Atlantic Forest of Diadema, Sao Paulo, Brazil. J Ethnobiol Ethnomed. 2010; 29: 6–29. 10.1186/1746-4269-6-29 PMC2987905 21034478

[18] Crepaldi MOS, Peixoto AL. Use and knowledge of plants by “Quilombolas” as subsidies for conservation efforts in an area of Atlantic Forest in Espírito Santo State, Brazil. Biodivers Conserv. 2010; 19: 1–37.

[19] Neto FRG, Almeida GSSA, Jesus NG, Fonseca MR. Estudo Etnobotânico de plantas medicinais utilizadas pela Comunidade do Sisal no município de Catu, Bahia, Brasil. Rev. Bras. Pl. Med. 2014; 16: 856–865.

[20] FUNDART. Quilombos. Prefeitura municipal de Ubatuba. 2014. https://fundart.com.br/tradicao/comunidades/quilombos/. Accessed 18 june 2018.

[21] ITESP. FUNDAÇÃO INSTITUTO TERRAS DE SÃO PAULO. Relatório técnico-científico sobre os remanescentes da comunidade de Quilombo de Camburi Ubatuba-SP. 2002. http://www.itesp.sp.gov.br/br/info/acoes/rtc/RTC_Cambury.pdf. Accessed 20 june 2018.

[22] Bernard HR. Research methods in cultural anthropology. Newbury Park, CA: Sage; 1988.

[23] Alexiades MN. Selected Guidelines for Ethnobotanical Research: A field manual. In: The New York Botanical Garden, New York; 1996.

[24] Malinowski B. Objeto, método e alcance desta pesquisa In: Desvendando máscaras sociais; 1990 p. 39–61.

[25] Grasser S, Schunko C, Vogl CR. Children as ethnobotanists: methods and local impact of a participatory research project with children on wild plant gathering in the Grosses Walsertal Biosphere Reserve. J Ethnobiol Ethnomed. 2016; 12: 46 10.1186/s13002-016-0119-6 27724928 PMC5057487

[26] Paniagua-Zambrana NY, Bussmann RW, Hart RE, Huanca ALM, Soria GO, Vaca MO, et al Who should conduct ethnobotanical studies? Effects of different interviewers in the case of the Chácobo Ethnobotany project, Beni, Bolivia. J Ethnobiol Ethnomed. 2018; 9: 14–1. 10.1186/s13002-018-0210-2 PMC5787299 29373988

[27] Stepp JR. Advances in ethnobiological field methods. Field Method. 2005; 17: 211–218.

[28] Hunn E. Ethnobiology in four phases. J Ethnobiol. 2007; 27:1–10.

[29] Wolverton S. Ethnobiology 5: interdisciplinaridade em uma era de rápidas mudanças ambientais. Cartas de Etnobiologia. 2013; 4: 21–5.

[30] Silva NCB, Regis ACD, Esquibel MA, Santos JES, Almeida MZ. Uso de plantas medicinais na comunidade quilombola da Barra II—Bahia, Brasil. BLACPMA. 2012; 11: 435–453.

[31] Beltreschi L, De Lima R, Da Cruz D. Traditional botanical knowledge of medicinal plants in a “quilombola” community in the Atlantic Forest of northeastern Brazil. Environment Development and Sustainabili. 2018; 21: 1185–1203.

[32] Rodrigues E. Plants and animals utilized as medicines in the Jaú National Park (JNP), Brazilian Amazon. Phytother Res. 2006; 20: 378–391. 10.1002/ptr.1866 16619367

[33] Santos JFL, Pagani E, Ramos J, Rodrigues E. Observations on the therapeutic practices of riverine communities of the Unini River, AM, Brazil. J Ethnopharmacol. 2012; 142: 503–515. 10.1016/j.jep.2012.05.027 22659194

[34] Rodrigues E, Carlini EA. Ritual use of plants with possible action on the central nervous system by the Kraho indians, Brazil. Phytother Res. 2005; 19: 129–135. 10.1002/ptr.1636 15852494

[35] Rodrigues E, Carlini EA. Plants used by a Quilombola group in Brazil with potential central nervous system effects. Phytother Res. 2004; 18: 748–753. 10.1002/ptr.1535 15478201

[36] Oliveira LR. Uso popular de plantas medicinais por mulheres da comunidade quilombola de furadinho em vitória da conquista, Bahia, Brasil. Revista Verde (Pombal—PB—Brasil). 2015; 10: 25–31.

[37] Moreira FR, Oliveira FQ. Levantamento de plantas medicinais e fitoterápicos utilizados na comunidade quilombola-pontinha de Paraopeba, Minas Gerais, Brasil. Rev. Bras. de Ciênc. da Vid. 2017; 5: 5.

[38] Zank S, Hanazaki N. The coexistence of traditional medicine and biomedicine: A study with local health experts in two Brazilian regions. PloS One. 2017; 12: 174–731. 10.1371/journal.pone.0174731 PMC5393556 28414735

[39] Kawa NC. How Religion, Race, and the Weedy Agency of Plants Shape Amazonian Home Gardens. Cult. Agric. Food and Env. 2016; 38: 84–93.

[40] Jovchelevich P, Câmara FLA. Influência dos ritmos lunares sobre o rendimento de cenoura (Daucus carota), em cultivo biodinâmico. Rev Bras Agroecol. 2008; 3: 1.

[41] Gomes TB, Bandeira FPSF. Uso e diversidade de plantas medicinais em uma comunidade quilombola no Raso da Catarina, Bahia. Acta Bot Bras. 2012; 26: 796–809.

[42] Tribess B, Pintarelli GM, Bini LA, Camargo A, Funez LA, De Gasper AL, et al Ethnobotanical study of plants used for therapeutic purposes in the Atlantic Forest region, Southern Brazil. J Ethnopharmacol. 2015; 164: 136–146. 10.1016/j.jep.2015.02.005 25680844

[43] De Santana BF, Voeks RA, Funch LS. Ethnomedicinal survey of a maroon community in Brazil's Atlantic tropical forest. J Ethnopharmacol. 2016; 181: 37–49. 10.1016/j.jep.2016.01.014 26802786

[44] Rego CARM, Rocha AE, De Oliveira CA, Pacheco FPF. Levantamento etnobotânico em comunidade tradicional do assentamento Pedra Suada, do município de Cachoeira Grande, Maranhão, Brasil. Ac Ag. 2016; 65: 284–291.

[45] Flora do Brasil 2020 em construção. Jardim Botânico do Rio de Janeiro. http://floradobrasil.jbrj.gov.br/. Accessed 06 september 2019.

[46] ICMBio (Instituto Chico Mendes Biodiversity Conservation). 2018. http://www.icmbio.gov.br/portal/. Accesed 12 december 2018.

[47] Martinelli G, Moraes MA. In: Livro vermelho da flora do Brasil. Rio de Janeiro: Instituto de Pesquisas Jardim Botânico do Rio de Janeiro; 2013.

[48] Almeida CFCBR, Albuquerque UP. Uso e conservação de plantas e animais medicinais no estado de Pernambuco (Nordeste do Brasil): um estudo de caso. Interciência. 2002; 26: 276–285.

[49] Voeks RA. Tropical forest healers and habitat preference. Econ Bot. 1996; 50: 381–400.

[50] Begossi A, Hanazaki N, Tamashiro JY. Medicinal Plants in the Atlantic Forest (Brazil): Knowledge, Use and Conservation Hum Ecol. 2002; 30: 281–299.

[51] Gazzaneo LRS, De Lucena RFP, Albuquerque UP. Knowledge and use of medicinal plants by local specialists in an region of Atlantic Forest in the state of Pernambuco (Northeastern Brazil). J Ethnobiol Ethnomed. 2005; 1: 1–9. 10.1186/1746-4269-1-9 PMC1291389 16270911

[52] Pinto E, Amorozo MCM, Furlan A. Conhecimento popular sobre plantas medicinais em comunidades rurais de mata atlântica–Itacaré, BA, Brasil. Acta Bot Bras. 2006; 20: 751–762.

[53] Amorozo MCM, Gély A. Uso de plantas medicinais por caboclos do baixo Amazonas. Barcarena, PA, Brasil. Bol Mus Para Emílio Goeldi Cien Biol. 1998; 4: 47–131.

[54] Albuquerque UP, Araújo TAS, Ramos MA, Nascimento VT, Lucena RFP, Monteiro JM, et al How ethnobotany can aid biodiversity conservation: reflections on investigations in the semi-arid region of NE Brazil. Biodivers Conserv. 2009; 18: 127–150.

[55] Tuler AC. Levantamento etnobotânico na comunidade rural de São José da Figueira, Durandé, MG, Brasil. Alegre: UFES. Trabalho de Conclusão de Curso apresentado ao Centro de Ciências Agrárias, Universidade Federal do Espírito Santo. 2011.

[56] Merzouki A, Ed-derfoufi F, Mesa JM. Contribution to the knowledge of Rifian traditional medicine. II: Folk medicine in Ksar Lakbir district (NW Morocco). Fitoterapia. 2000; 71: 278–307. 10.1016/s0367-326x(00)00139-8 10844168

[57] Garlet B, Irgang B. Plantas medicinais utilizadas na medicina popular por mulheres trabalhadoras rurais de Cruz Alta, Rio Grande do Sul, Brasil. Rev Bras Pl Med. 2001; 4: 9–18.

